# Circular economy transition barriers in the construction and demolition sector

**DOI:** 10.1371/journal.pone.0338631

**Published:** 2025-12-11

**Authors:** Majed Alzara, Ahmed Abd Elnasser, Mohammed Abdelshakor, Ahmed Ehab

**Affiliations:** 1 Department of Civil Engineering, College of Engineering, Jouf University, Sakaka, Saudi Arabia; 2 Department of Civil Engineering, Arab Academy for Science, Technology and Maritime Transport, Aswan, Aswan Governorate, Egypt; 3 Department of Civil Engineering, College of Engineering, Badr University in Cairo, Cairo, Egypt; Kwame Nkrumah University of Science and Technology, GHANA

## Abstract

This study evaluates the readiness of Egypt’s construction sector to integrate Circular Economy (CE) principles by identifying and analyzing the main barriers to effective Construction and Demolition Waste (CDW) management. A comprehensive survey-based methodology (n = 384) was employed to gather data from industry stakeholders, demonstrating excellent sampling adequacy (KMO = 0.961). Initially, 41 potential barriers were identified via literature review and expert consultation. Exploratory Factor Analysis (EFA) subsequently refined these to 10 critical barriers, whose interrelationships were validated using Structural Equation Modeling (SEM), exhibiting a strong model fit (RMSEA = 0.065; CFI = 0.974). The analysis revealed persistent economic, technological, and social barriers impeding CE adoption. Notable challenges include high implementation costs, inadequate technical expertise, low stakeholder awareness, and weak policy enforcement. Based on these findings, prioritized, time-phased recommendations are proposed to provide an evidence-based framework for policy and practice. This research contributes an early empirical assessment of CE adoption in Egypt’s construction sector, offering a robust roadmap for a more sustainable, resource-efficient industry aligned with national environmental and economic objectives.

## 1. Introduction

The construction industry is a key part of modern cities, fueling economic growth and urban development worldwide. However, it is also one of the largest sources of waste, accounting for about 30% of global waste [1,2]. This significant waste production emphasizes the urgent need for more sustainable industry practices. Adopting CE principles provides a transformative approach to these challenges. CE fosters a closed-loop system where resources are reused, recycled, and regenerated continuously, moving away from the conventional linear “take-make-dispose” model that causes excessive waste and environmental harm [3]. Recently, the concept of a CE has gained significant momentum, with a 21% annual increase in related construction sector publications [4]. This growing interest highlights CE’s potential to lessen environmental impacts while boosting economic efficiency and innovation. However, adopting CE in the construction and demolition sectors, especially in developing countries like Egypt, faces notable obstacles [5]. The industry’s impact on solid waste, raw material extraction, and greenhouse gas emissions calls for a shift toward sustainable waste management practices. Three main areas—social, economic, and environmental—must be prioritized to overcome the challenges facing CE. Although CE principles have gained global traction in the construction sector, significant research gaps persist, particularly in developing countries like Egypt. Several influential studies that propose frameworks or empirically analyze barriers to CE in the built environment have been developed in developed-country contexts. For instance, Giorgi et al. and Kanters [6,7] analyze European settings and derive policy-oriented frameworks tailored to those institutional landscapes; Aslam et al. and Ghisellini et al [8] review barriers primarily using data and cases from China, the UK, and other high-income settings. These frameworks are valuable but may not fully account for governance fragmentation, informal market dynamics, and subsidy structures that characterize many developing economies such as Egypt. Therefore, applying or directly transferring such frameworks to Egypt risks overlooking context-specific socio-economic and regulatory constraints that this study seeks to address. Most existing studies are concentrated in developed contexts, offering frameworks that may not suit Egypt’s unique socio-economic and regulatory environment. Moreover, while CDW contributes substantially to global waste, its management within Egypt’s construction industry remains underexplored. Current literature often isolates economic, social, or technological barriers to CE adoption, lacking an integrated approach that captures their interdependencies. There is also a shortage of empirical studies using robust methodologies—such as Exploratory Factor Analysis (EFA) and Structural Equation Modeling (SEM)—to uncover latent barriers and causal relationships. Additionally, strategic policy guidance tailored to Egypt’s construction sector is limited, leaving stakeholders without actionable insights for CE implementation. Finally, few studies incorporate the perspectives of key actors like contractors, engineers, and investors, whose engagement is crucial for driving sustainable transformation. Addressing these gaps is essential to advancing CE adoption and sustainable waste management in Egypt’s construction industry.

Egypt is one of the developed countries, particularly in the construction sector. Therefore, the management of CDW is necessary and contributes a positive impact on sustainable management. On the other hand, the slackness of this point will exacerbate the crisis of the environment. Also in Egypt, economic, technological, and social factors are the main categories of the challenges and barriers facing the adoption of CDW.

This study aims to identify and analyze the barriers to implementing CE practices in the Egyptian construction sector. Utilizing a mixed-method approach that combines Exploratory Factor Analysis (EFA) and Structural Equation Modeling (SEM), this research seeks to offer a detailed understanding of these barriers and provide actionable recommendations for their mitigation. This study’s findings will guide policymakers, industry stakeholders, and financial investors in fostering sustainable construction practices and advancing the transition towards a CE in Egypt. Ultimately, it will significantly contribute to the academic discourse on sustainable waste management and offer practical solutions for enhancing CE adoption in the Egyptian construction industry. By addressing the identified barriers, this research strives to pave the way for a more sustainable future, characterized by reduced environmental impacts and improved economic resilience.

## 2. Literature review

The construction industry is a major contributor to global waste, accounting for about 30% of all waste produced [1]. In response, the CE has emerged as a transformative approach aimed at reducing waste, conserving resources, and promoting sustainability. Unlike the traditional linear model of “take-make-dispose,” CE emphasizes a closed-loop system that focuses on reuse, recycling, and regeneration [2,3]. Recent studies show a growing interest in CE within the construction industry, with CE-related publications increasing by 21% each year [4]. This growth reflects a broader awareness of the environmental and economic challenges caused by CDW, including resource depletion, greenhouse gas emissions, and landfill overuse [5,9].

The CE offers a promising way to reduce environmental impacts and improve sustainability in the construction sector. Currently, the CDW sector significantly contributes to solid waste, raw material extraction, and greenhouse gas emissions. These impacts can be minimized by shifting from a linear production model to a CE and increasing the reuse of building materials. As noted in [9], there is growing interest in adopting CE principles, such as reducing, reusing, and recycling materials, to better manage construction waste. Addressing the environmental challenges posed by CDW requires considering social, economic, environmental, spatial, and temporal factors [10]. The sector’s environmental impacts—such as resource consumption, emissions, and pollution—highlight the urgency for change and the importance of sustainable waste management practices [11]. To tackle these challenges, the idea of a CE has gained momentum within the construction industry. This approach promotes the principles of reduce, reuse, and recycle, which aim to minimize waste and maximize resource efficiency. Implementing these principles allows the CDW sector to not only contribute to developing sustainable engineering and CE but also to cut its own environmental impacts.

The most common way to reduce the environmental impacts of the CDW sector is by adopting alternative construction methods [12]. Decision-makers are recognizing the importance of using these methods to cut waste and improve resource management. Alternative construction methods can include using sustainable materials and systems where materials are reused, reduced, recycled, or repurposed instead of being thrown away as CDW [12]. Therefore, to maximize the benefits and minimize the challenges of implementing a CE, individuals, businesses, governments, and communities can all play vital roles.

By taking a proactive role in supporting and advancing the principles of a CE, individuals, businesses, governments, and communities can collectively work to maximize its benefits while minimizing challenges and contributing to a more sustainable and resilient future [13].

On the other hand, the emerging models within the CE framework, specifically applied to the CDW sector, present innovative paradigms aiming to revolutionize waste management practices. These models advocate for a systemic shift from the traditional linear “take-make-dispose” approach towards a more regenerative and sustainable system, emphasizing waste reduction, reuse, recycling, and resource recovery. However, within the complex landscape of the construction industry, the implementation of these emerging models encounters multifaceted barriers. Challenges such as the fragmented nature of the construction supply chain, the lack of standardized practices, and the complexities inherent in materials and building composition pose significant hurdles. Additionally, limited awareness, financial constraints, inadequate infrastructure, and regulatory inconsistencies hinder the seamless integration of CE models into CDW management. Overcoming these barriers necessitates a concerted effort involving stakeholders across the construction spectrum, the establishment of standardized protocols, supportive policies, enhanced collaboration, technological innovations, and increased investment in research and development. These efforts are vital to catalyze the transformation towards circularity in the CDW sector of the construction industry, fostering sustainable waste management practices and resource optimization. From the previous works and literature review, 41 barriers that affect the implementation of the CE in the construction and demolition sector are determined. Then, some experts from the construction industry with experience exceeding 10 years and using semi-structured interviews, reviewed the collected barriers. After adding, removing, and combining similar factors related to the experts’ visions in the “Egyptian construction industry environment,” about 41 barriers were identified for further analysis, as shown in Table 1. Then, a filtration process for these barriers was done again by a new survey with owners, managers, engineers, and supervisors as construction professionals participating. This survey identifies the most important factors that affect the implementation of a CE in the construction and demolition sector in Egypt. To organize these barriers, five main work packages are created to classify all these barriers and arrange each barrier under its work package. The next sections are briefs for these work packages.

**Table 1:** Barriers affecting the implementation of CE in the construction and demolition sector [13].

Code	Barrier	Source
B1	Robust availability and low price of raw materials.	[13] and [8]
B2	A lack of economic mechanisms for The reuse of materials.	[14]
B3	exorbitant expenses of disassembly, segregation, processing, shipping, and storage of CDW.	[15] and [14]
B4	Expensive rates of recycled products.	[8]
B5	Absence of incentive and sanction systems for CDW management operations.	[16] and [15]
B6	Product prices do not reflect environmental costs. account.	[13]
B7	Financial and risk avoidance for circular business models.	[17]
B8	A culture of speedy gains on investment and elevated costs for green buildings.	[18] and [19]
B9	Expense of developing product certifications.	[20] [21]
B10	Poor quality of recycled and reused material.	[15]
B11	Expensive capital outlay for waste technologies.	[22] and [8]
B12	Negative community perception (due to lack of communication, trust, and awareness).	[15] and [8]
B13	Social and behavioral dimensions of contemporary consumerism.	[22] and [23]
B14	Shortage of promotion and educational campaigns.	[15]
B15	Minimal environmental conservation programs and facilities at academic institutions.	[23]
B16	Conventional, competitive, and disjointed supply chains.	[23] and [8]
B17	Insufficient knowledge about DFD, eco-friendly design, and end-of-life products.	[24] and [21]
B18	Limited understanding of circular tools (EPDs, Material Passports, certifications, etc.).	[16] and [20]
B19	Limited implementation of the waste hierarchy (with excessive focus on recycling).	[8] and [25]
B20	Once of guidance and tools for the implementation/ Assessment of circular buildings.	[17]
B21	Absence of incentive and facilitation for end-of-life design (limited focus on DFD).	[13] and [14]
B22	Rigidity in building codes and regulations.	[6]
B23	Absence of standardized EPD (Environmental Product Declaration) at an international level.	[13] and [20]
B24	Insufficiency of producer-based responsibility system and regulatory framework for integrated resource management.	[23] and [25]
B25	Inadequate waste code to govern CDWM and discourage landfilling.	[8]
B26	Insufficiency of a tax regime and standard quality for reclaimed water materials.	[26] and [23]
B27	Lack of regulations mandating to assignment of a minimum percentage of CDW for reusing and recycling.	[8]
B28	Lack of land-use zoning and rational urban planning.	[23]
B29	Lack of national objectives, targets, and a legal support system with a binding effect.	[17] and [8]
B30	Limited assistance for research, innovation, information, and business procurement strategies.	[17] and [26]
B31	Inadequate oversight from the government (with qualified professionals and budget constraints).	[23] and [8]
B32	Poor CDW management.	[7] and [6]
B33	Recycling practices are hindered by the limited separation of materials, logistical barriers, and a lack of process to produce easily disassembled products.	[23] and [7]
B34	Lack of tools for identifying, classifying, and certifying of salvaged materials.	[21] and [8]
B35	Complicated material composition and building composition (numerous layers and modifications during its lifespan).	[27] and [26]
B36	Lack of standardized spatial geometries and limited visualization for DFD.	[27] and [21]
B37	Ineffective development of sustainable building designs.	[19]
B38	Inadequate data quality and accessibility (concerns about privacy, trust, ownership, and access).	[23] and [22]
B39	Difficulties in understanding and developing EPDs.	[20]
B40	Absence of documentation for new and used building products.	[22]
B41	Scarcity of datasets and tools compliant with BIM.	[21] and [28]

### 2.1. Economic barriers

The Implementation of a CE in the CDW sector poses several challenges [29]. These challenges include economic factors, such as high availability and low costs of virgin raw materials, as well as the need for sustainable, recyclable, repairable products and materials [10]. In addition, there are economic barriers that include the lack of economic benefits associated with sorting and recycling, as well as the need for incentives and value creation to encourage CE practices. On the other hand, effective marketing strategies to promote sustainable practices in the CDW sector are in focus these days. In addition, the CDW sector is facing some challenges, which may result in cost and time overruns of the project and waste energy. In addition, related economic factors as the high prices of recycled or reused materials and products within the construction industry, coupled with the absence of effective reward and penalty schemes for (CDW) management operations, present formidable barriers to the widespread adoption of CE principles [8]. Furthermore, the absence of comprehensive reward and penalty schemes for CDW management operations exacerbates the situation. Without adequate incentives or repercussions for sustainable waste management practices, stakeholders may lack the motivation to invest in efficient waste sorting, recycling, or reuse strategies. The absence of clear rewards or penalties fails to internalize the environmental and social costs associated with improper waste disposal, hindering the transition towards more responsible waste management practices. [15].

### 2.2. Informational barriers

Social and behavioral aspects inherent in modern consumerism play a crucial role in shaping public perception and attitudes toward sustainability. Consumer preferences often prioritize convenience, cost-effectiveness, and immediate gratification over long-term environmental impacts, influencing demand for construction materials and building practices. This consumer-centric mindset, characterized by a focus on short-term benefits and a lack of emphasis on sustainable choices, further impedes the adoption of CE principles within the construction industry [22]. Furthermore, the absence of effective publicity and information campaigns targeted at educating and engaging the public exacerbates the issue [15]. Limited efforts to raise awareness, inform, and mobilize stakeholders, including consumers, businesses, and policymakers, hinder the uptake of sustainable construction practices. Lastly, academic institutions’ insufficient emphasis on environmental management programs and facilities contributes to the challenge. Inadequate integration of sustainability education and research within academic curricula limits the cultivation of future professionals’ awareness and understanding of CE principles, thereby affecting their ability to drive change within the industry [23].

### 2.3. Institutional Barriers

The construction industry grapples with multifaceted challenges hindering the widespread adoption of CE principles, notably stemming from conservative, competitive, and fragmented supply chains. These supply chains, often entrenched in traditional practices and characterized by stiff competition among stakeholders, create barriers to the implementation of circularity. Conservative attitudes within supply chains tend to resist change, maintaining a status quo that favors linear processes and conventional material sourcing [23]. In addition, the missing understanding of the green design concept among designers and stakeholders affects the environment and prevents the integration of sustainable design strategies that minimize environmental impact throughout a building’s lifecycle [30].

### 2.4. Political Barriers

The lack of incentives and support to design for end-of-life scenarios, underscored by low prioritization for Design for Disassembly (DfD) strategies, represents a substantial impediment to the adoption of CE principles within the construction industry. DfD methodologies, aimed at facilitating easier disassembly and material recovery at a building’s end-of-life, often face limited acknowledgment and implementation due to the absence of adequate incentives [21]. Additionally, the rigidity and lack of flexibility in building codes and regulations further constrain the integration of innovative and sustainable design practices. These regulations often prioritize conventional construction methods and materials, impeding the incorporation of DfD principles and inhibiting the evolution towards more circular building practices [6]. Moreover, the lack of standardized Environmental Product Declarations (EPDs) on an international level hampers informed decision-making regarding material selection and resource management. The absence of a globally recognized and standardized EPD framework restricts the comparability and reliability of environmental information on construction materials, hindering efforts to promote sustainable material choices and circularity [31].

### 2.5. Technological barriers

The ineffective management of CDW presents a significant impediment to the advancement of CE principles within the construction industry. Recycling practices encounter substantial hurdles due to limited material separation, logistical complexities, and the absence of processes to manufacture easily disassembled products. The absence of tools for identifying, classifying, and certifying salvaged materials complicates their integration into the supply chain. The lack of standardized methodologies and certification mechanisms for salvaged materials limits their market acceptance and utilization, impeding efforts to promote their reuse or recycling [26]. Furthermore, the complexity of materials and building composition, characterized by multiple layers and modifications throughout their lifespan, poses a formidable challenge. The intricate nature of building materials, including composite structures and mixed materials, complicates the disassembly and recycling processes, making it challenging to recover resources effectively [27].

## 3. Research methodology

This research employs a mixed approach, beginning with the compilation and analysis of existing literature to identify barriers that influence the CE in the CDW sector. Therefore, the coming steps are the main process used in this research to achieve the goals of the research:

The first step is to identify the most significant factors among those identified in the literature by a survey carried out in Egypt. Various methods, including interviews, postal, and electronic surveys utilized for data collection.

An initial qualitative phase was conducted to refine and contextualize barriers identified in the literature. We carried out semi-structured interviews with 12 domain experts who possess extensive experience (>10 years) in Egypt’s construction and CDW management. The expert sample included 6 senior contractors/project managers, 3 consulting engineers/CE specialists, and 3 public-sector officials (waste management/policy). Interviews lasted 40–60 minutes and followed a semi-structured guide focusing on (a) perceived barriers to CE adoption, (b) contextual constraints specific to Egyptian construction practice, and (c) recommended priority interventions. Interview data were coded thematically; experts validated, merged, or recommended additions to the initial list compiled from the literature. This process produced a context-adjusted list of 41 candidate barriers used in the subsequent survey-based quantitative phase.

To identify the barriers that deeply affect the implementation of (CE) in the CDW sector in the Egyptian environment, and to make a suitable model for Egypt’s environment, a questionnaire survey has been created. The survey consists of two parts: the first mandatory part of the questionnaire that designed to collect general data about the survey participants such as job title (owner, consultant, and contractor), experience years in the construction industry, and the participant’s knowledge about CE) in the CDW sector were asked of the respondents. In the second part, a list of the barriers affecting the adoption and empowerment of CE in the construction and building industry is presented in Table 1. To measure the degree of impact on the CDW sector, a qualitative analysis is created to rate each factor using a 1–5 Likert scale, where “1” means strongly disagree, “2” disagree, “3” moderate, “4” agree, and “5” strongly agree. The questionnaire covered Cairo, the 10^th^ of Ramadan, 6 October, Assiut, Aswan, Alexandria, and Giza in Egypt, related to large construction projects in these cities today.

This study was approved by the research ethics committees of both Badr University in Cairo and the Arab Academy for Science, Technology and Maritime Transport in Alexandria. All participants were adults (aged 18 and above) and provided informed consent prior to their participation. consent was obtained in written form via email, and participants were clearly informed of the study’s objectives, procedures, voluntary nature, and their right to withdraw at any stage without penalty.

This research mainly focused on the construction and building sector and its branches (facility works, construction of metal works, foundation works, and specialized complementary works). The target sample of this analysis is the construction companies that are classified into seven grades (depending on their size) related to the classification of the Egyptian Federation of Building and Construction Contractors (EFBCC) [32] into seven ranks. These ranks ascend from the first grade (for big size of combines) to the seventh grade (for tiny size of combines). Related to [32], in 2024, the number of civil engineers was 858,000, related to the statistics issued by the Egyptian Engineers Syndicate’s registry. For the reliability and credibility of the survey and identification, the proper sample size is important. There will be a loss of significant study findings if the sample size is too small, with an acceptable margin of error equal to 5% and a confidence level equal to 95%. On the other hand, the Kaiser-Meyer-Olkin (KMO) test (Eq. 1) is implemented to measure the adequacy of the sample size. In other words, the suitability of the data for analysis [33].


$$KMO=\frac{\sum_{i\neq j}R_{ij}^{2}}{\sum_{i\neq j}R_{ij}^{2}+\sum_{i\neq j}U_{ij}^{2}}$$ (1)

Where: $R_{ij}$ is the correlation matrix, and $U_{ij}$ is the partial covariance matrix, and the sample evaluation related to its KMO number must be under consideration as the following [34], when KMO value within (0.8–1.0) then the sample evaluation become “Adequate”, when KMO value within (0.7–0.79) then the sample evaluation become “Middle”, when KMO value within (0.6–0.69) then the sample evaluation become “Mediocre”, Finally when KMO less than 0.6 then the sample evaluation become “ Not adequate”. For this research, the value of the KMO statistic is 0.961, which means the sample size (384 people) is adequate.

The sample composition (42% contractors, 30% consultants, 26% owners/public officials; experience distribution skewed toward mid-senior levels) provides a practitioner-centered view of CE barriers in major project contexts. The predominance of contractor and consultant voices suggests the findings chiefly reflect operational and procurement perspectives, while the lower representation of policymakers indicates the need for targeted follow-up engagement with regulatory stakeholders. The distribution of CE awareness also signals that while knowledge exists among many practitioners, gaps remain — supporting our emphasis on awareness and capacity-building interventions.

**Fig 1:**
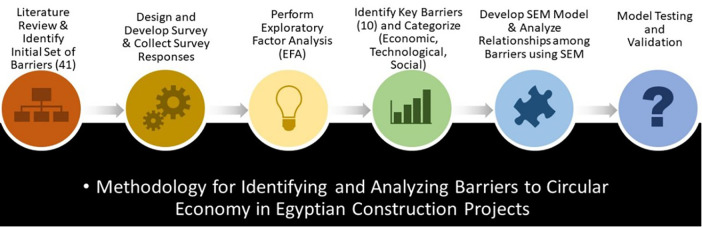
Sequence Steps and the Methodology of the Research.

The second step is analyzing the survey and determining the loading threshold of the barrier, that done by the exploratory factor analysis. Exploratory factor analysis is the first step used to categorize factors, prioritize the determined barriers related to the collected responses, and analyze them. Then, using the structural equation model (SEM) to determine the weights of the most important group of factors to develop a model and examine relationships among observed and latent variables. Sequence, developing an index for evaluating the CE implementation. Finally, the developed model is tested and validated. Fig 1 presents the sequential steps, each of which builds upon the previous one, leading to the development of a comprehensive framework for the research topic.

### 3.1. Exploratory Factor Analysis (EFA).

EFA on of the most common statistical data analysis techniques for prioritizing factors in data sets and determining the consistency of one another. Also, highlights the importance of weight from a list of factors [35]. Statistical Package for Social Science (SPSS) is the method used in this research as a quantitative method. A matrix of correlations among factors is created in the first step of the analysis to show the Pearson correlation coefficients for each pair of factors. The factors that should be under consideration have correlations greater than 0.3 but less than 0.9, related to [36]. The factors that have a correlation range of < 0.3 will be ignored and not considered unimportant. Oppositely, it means this factor is highly correlated when the correlation is > 0.9. By applying the Bartlett Test of Sphericity, the population correlation matrix is not an identity matrix, which has a result of 820.00 and a significance level of zero. All of the results from the Bartlett Test of Sphericity and the Kaiser-Meyer-Olkin (KMO) will be under consideration in the EFA and the possibility of condensing these factors into a smaller set [34]. The result of this analysis is identifying the barriers associated with preventing the adoption and empowerment of the CE in the construction and building industry in Egypt. This analysis gives credibility and adds value to the surveying data to delve deeply into the main dimensions of this study and its relationships.

### 3.2. Result of Exploratory Factor Analysis (EFA).

The results of the EFA show that Factor 1 is consistent with barriers B1, B2, B3, and B7 strongly, with weights of 0.763, 0.745, 0.736, and 0.700, respectively. Similarly, Factor 2 engagement with barriers B38, B37, B36, and B39, with weights of 0.733, 0.727, 0.721, and 0.704, respectively. Also, the high Factor 3 is linked with B31 and B27, indicating their strong relationship with this factor (0.742 and 0.712, respectively). The minimum limit of the loading criterion at the EFA analysis is 0.7. So, the variables that did not satisfy this criterion were excluded from further analysis. Therefore, thirty-one barriers (the remaining barriers, excluding the ones mentioned above) are omitted from the factor solution as shown in Table 2. Maximize the variance of each factor and create a factor structure by using a Varimax rotation.

**Table 2:** Analysis of the Exploratory Factor.

Variable	1	2	3
B1	0.763		
B2	0.745		
B3	0.736		
B7	0.700		
B38		0.733	
B37		0.727	
B36		0.721	
B39		0.704	
B31			0.742
B27			0.712
Extraction	B4, B5, B6, B8, B9, B10, B11, B12, B13, B14, B15, B16, B17, B18, B19, B20, B21, B22, B23, B24, B25, B26, B28, B29, B30, B32, B33, B34, B35, B40, B41		

Finally, Factors 2 and 3 are identified as the most important ones related to the maximum loadings on each factor, signifying their strong association with the respective factor. Table 3 shows the final categorization of the variables; the variables are also distributed into three main groups.

**Table 3:** Categorization of variables after extraction from EFA analysis.

*Category*	*Code*	*Barrier*
*Economic barriers*	*B1*	Robust availability and low price of raw materials.
	*B2*	Lack of economic mechanisms for the reuse of materials.
	*B3*	Exorbitant expenses of disassembly, segregation, processing, shipping, and storage of CDW.
	*B7*	Financial and risk avoidance for circular business models.
*Technological barriers*	*B36*	Lack of standardized spatial geometries and limited visualization for DFD.
	*B37*	Ineffective development of sustainable building designs.
	*B38*	Inadequate data quality and accessibility (concerns about privacy, trust, ownership, and access).
	*B39*	Difficulties in understanding and developing EPDs.
*Social barriers*	*B27*	Lack of regulations mandating to assignment of a minimum percentage of CDW for reusing and recycling.
	*B31*	Inadequate oversight from the government (with qualified professionals and budget constraints).

To preserve nuance, excluded items are documented in Table 2; ten retained barriers represent statistically robust cluster centroids reflecting primary dimensions (economic, technological, social). Selection used a loading threshold of 0.70 and theoretical interpretability; thus, the reduction thereby emphasizes parsimony without discarding important secondary issues altogether.

### 3.3. Structural Equation Modeling (SEM).

Structural Equation Modeling (SEM) is a robust statistical technique for assessing complex relationships among observed and latent variables [37]. It integrates both measurement and structural models to test theoretical frameworks, allowing researchers to examine direct and indirect effects simultaneously. SEM offers advantages over traditional regression analysis by accommodating multiple dependent variables and assessing measurement error. Additionally, it provides valuable insights into the underlying structure of relationships within a model. Notably, SEM has been widely applied across various disciplines, including psychology, sociology, neuropsychology, and engineering studies [38].

### 3.4. Result of Structural Equation Modeling (SEM).

Factor Loadings (e.g., 0.69, 0.8, 0.84, 0.68, 0.82, 0.86, 0.85, 0.77, 0.75, and 0.79) represent the correlation between an observed variable and a latent variable. They indicate how well the observed variables measure the latent constructs. A higher factor loading indicates a stronger relationship between the observed variable and the latent construct. For example, a factor loading of 0.75 means that the observed variable has a strong correlation with the latent variable it is supposed to measure. Generally, factor loadings above 0.70 are considered strong, indicating that the observed variable is a good indicator of the latent variable. Loadings between 0.50 and 0.70 are moderate, and those below 0.50 are weak.

Path Coefficients between Latent Variables (e.g., 0.5, 0.54, 0.77). These coefficients represent the strength and direction of the relationship between two latent variables. A positive coefficient indicates a direct relationship, where an increase in one latent variable leads to an increase in the other. A negative coefficient indicates an inverse relationship. The closer the coefficient is to 1 or −1, the stronger the relationship.

**Fig 2:**
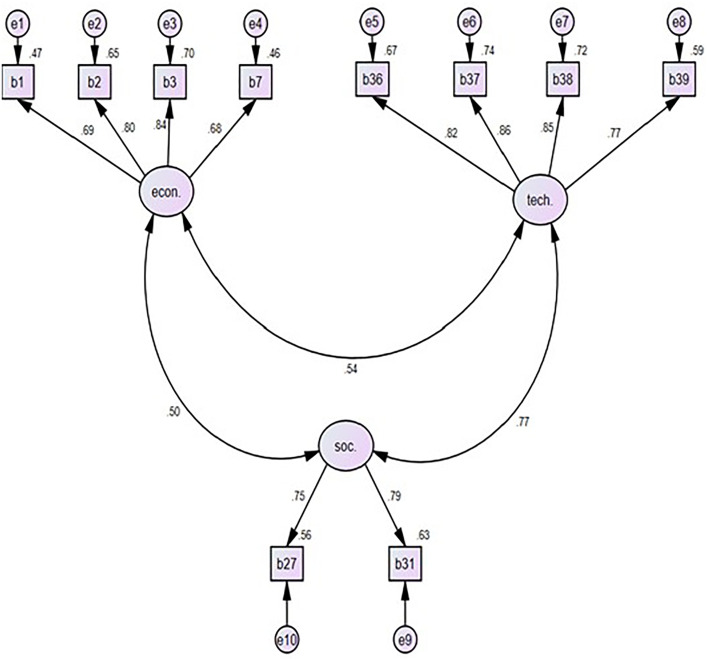
Result of structural equation modeling (SEM) analysis.

In SEM, each observed variable (survey item) is assumed to have some degree of measurement error. These errors are denoted as (e1, e2, e3, etc.). These numbers represent the variance of the measurement errors for each observed variable. A higher number indicates a higher amount of unexplained variance, meaning the observed variable has a greater measurement error. Fig 2. Shows the results of the SEM of the research. These SEM relationships theoretically confirm that economic, regulatory, and technological barriers interact as interdependent constructs influencing CE adoption. Practically, this implies that policy interventions targeting one barrier type may indirectly mitigate others, highlighting the systemic nature of CE transition challenges in Egypt’s construction sector.

The recommendation to introduce financial incentives is grounded in the strong statistical prominence of economic items: B1 and B3 recorded the highest factor loadings in the EFA (0.76 and 0.74, respectively), and the SEM indicates that economic barriers exerted a significant direct effect on CE adoption readiness (standardized path coefficient β = 0.62, p < 0.001). Therefore, fiscal instruments directly target the construct identified as the most influential.

- Model fit

Model fit indices such as Chi-Square Minimum (CMIN), Root Mean Square Residual (RMR), Goodness of Fit Index (GFI), Baseline Comparisons (CFI), Non-Centrality Parameter (NCP), Fit Function Minimum (FMIN), Root Mean Square Error of Approximation (RMSEA), Akaike Information Criterion (AIC), Expected cross-validation index (ECVI), and Holter assess the goodness-of-fit of a statistical model. They balance model accuracy and complexity, ensuring robust and reliable conclusions from the data. Tables 4 and 5 show all the calculations for all these parameters and more, focusing on the case study.

**Table 4:** Calculations for (CMIN, RMR, GFI, CFI, NCP, FMIN, and others) parameters related to the case study.

Model	NPAR	CMIN	DF	P	CMIN/DF	RMR	GFI	AGFI	PGFI	NFI Delta1	RFI rho1	IFI Delta2	TLI rho2	CFI	PRATIO 1	PNFI	PCFI	NCP	LO 90	HI 90	FMIN	F0
Chi-square and fit indices																						
Default model	23	82.215	32	.000	2.569																	
Saturated model	55	.000	0																			
Independence model	10	1951.615	45	.000	43.369																	
Root Mean Square Residual (RMR) and Goodness of Fit Index (GFI)																						
Default model						.027	.957	.927	.557													
Saturated model						.000	1.000															
Independence model						.345	.343	.197	.281													
Model A: No Autocorrelation						.284	.975	.913	.279													
Model B: Most General						.757	.998	.990	.238													
Baseline comparison: Incremental fit indices																						
Default model										.958	.941	.974	.963	.974								
Saturated model										1.000		1.000		1.000								
Independence model										.000	.000	.000	.000	.000								
Parsimony-Adjusted Measures																						
Default model															.711	.681	.692					
Saturated model															.000	.000	.000					
Independence model															1.000	.000	.000					
Non-Centrality Parameter (NCP)																						
Default model																		50.215	27.197	80.910		
Saturated model																		.000	.000	.000		
Independence model																		1906.615	1765.754	2054.831		
Fit Function Minimum (FMIN)																						
Default model																			.074	.221	.225	.137
Saturated model																			.000	.000	.000	.000
Independence model																			4.824	5.614	5.332	5.209

**Table 5:** Calculations for (RMSEA, AIC, ECVI, and Holter) parameters focusing on the case study.

Model	RMSEA	LO 90	HI 90	PCLOSE	LO 90	AIC	BCC	BIC	CAIC	ECVI	LO 90	HI 90	MECVI	ECVI	HOELTER .05	HOELTER .01
Root mean square error of approximation (RMSEA)																
Default model	.065	.048	.083	.069	.065											
Independence model	.340	.327	.353	.000	.340											
Akaike Information Criterion (AIC)																
Default model						128.215	129.641	218.039	241.039							
Saturated model						110.000	113.408	324.795	379.795							
Independence model						1971.615	1972.235	2010.669	2020.669							
Expected Cross-Validation Index (ECVI)																
Default model											.350	.287	.434	.354		
Saturated model											.301	.301	.301	.310		
Independence model											5.387	5.002	5.792	5.389		
HOELTER																
Default model															206	239
Independence model															12	14
Model A: No Autocorrelation															164	219
Model B: Most General															1615	2201

- **Chi-Square Minimum (CMIN)**

Researchers have suggested the use of this ratio as a measure of fit. For every estimation criterion except for Uls and Sls, the ratio should be close to one for correct models [39]. Suggests that the researcher also compute a relative chi-square. However, degrees of freedom ratios in the range of 3–1 are indicative of an acceptable fit between the hypothetical model and the sample data [40]. The data needed for this ratio is related to [41].

- **Root Mean Square Residual (RMR) and Goodness of Fit Index (GFI)**

The RMR (root mean square residual) is known as the square root of the average squared amount by which the sample variances and covariance differ from their estimates obtained under the assumption that the model is correct [40].

- **Baseline Comparisons (CFI)**

The CFI is a factor in calculating the relativity of the non-centrality with a range (0–1). When the CFI is close to one, it means “very good fit,” and the opposite is if it is close to zero.

- **Parsimony-Adjusted Measures**

Related to [42] & [43], the Parsimony Ratio is the ratio between the number of constraints in the model and the number of constraints in the independence mode. Also, to determine the number of degrees of freedom for different models (evaluated or baseline) by multiplying a Parsimony index by NFI and GFI.

- **Non-Centrality Parameter (NCP)**

NCP stands for Non-Centrality Parameter. The non-centrality parameter directly influences the power of a statistical test.

- **Fit Function Minimum (FMIN)**

FMIN can represent the optimal solution achieved by minimizing the error or discrepancy between a model and observed data, providing insights into the goodness of fit or the effectiveness of the model in capturing the underlying relationships in the data.

- **Root Mean Square Error of Approximation (RMSEA)**

RMSEA is an indicator of the fit of the solution to the problems with multiple values and different indications. Therefore, if RMSEA is close to or less than 0.05, this means that the model is a close fit in concerning of the degrees of freedom. Also, if RMSEA is about or less than 0.08, it indicates a reasonable error of approximation. Finally, it means not wanting to employ a model with an RMSEA greater than 0.1. [44].

- **Akaike Information Criterion (AIC)**

The Akaike information criterion (AIC) is a widely used statistical measure for model selection. It was proposed by Hirotugu Akaike in his seminal paper in 1974 and later refined in 1987 [45]. The AIC balances the goodness of fit of a model with its complexity, providing a tool for comparing different models based on their ability to explain the data while penalizing excessive complexity.

- **Expected Cross-Validation Index (ECVI)**

This index is so close to the previous index, called the Akaike Information Criterion. In addition, the confidence interval of the population with low and high limits (90%) must be determined.

- **HOELTER**

Hoelter’s critical value is utilized to determine the acceptability of a factor analysis solution.

## 4. Results and discussion

**Fig 3:**
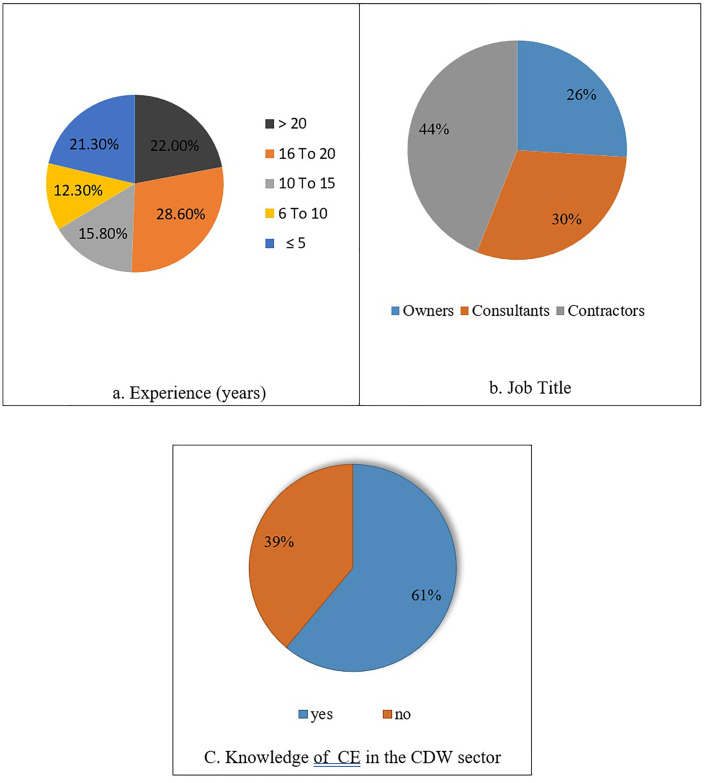
Survey respondents’ demographics.

The analysis of the questionnaire illustrates the participants’ responses by demographics as shown in Fig 3 below. The results show, about 22% of the respondents were experts for more than 20 years, 28.6% had about 16–20 years of experience in the construction field, 15.8% for those have experience of 10–15 years, 12.30% had 6–10 years of experience, and about 21.30% less than or equal to five years of experience. Also, the respondents were classified related to their role for each one in the different projects, with about 26% as owners, 30% consultants, and 44% simulated as contractors. Finally, they also divided into 61% of the respondents knew about the CE, while the remaining didn’t have anything about it.

The factor analysis and SEM results indicate that economic barriers occupy a primary position in constraining CE adoption in Egypt’s CDW sector. These economic constraints—characterized by the availability and low price of virgin materials, high upfront costs for recycling infrastructure, and perceived financial risks of circular business models—create a market environment where circular options are often less competitive. Consequently, a policy package focused on fiscal incentives (subsidies, tax exemptions for recycled-content products), blended finance to de-risk circular ventures, and support for shared waste-handling infrastructure (to achieve economies of scale) emerges as a priority. These recommendations follow directly from the high factor loadings and from expert interview narratives emphasizing cost as a recurrent barrier.

Technological and informational constraints form the second cluster: data quality and accessibility, limited tools for identifying and certifying salvaged materials, and weak digital/material traceability. These limitations impede the creation of transparent secondary-material markets. Responses therefore require investments in interoperable data platforms (linked to BIM practices), standards for material passports and EPDs, and capacity-building programs to raise technical competency in DfD (Design for Disassembly) and material reuse. The expert interviews specifically highlighted the role of digital traceability as a near-term enabler for market acceptance of reclaimed materials.

The social and institutional cluster (including inadequate regulatory oversight, absence of mandatory reuse targets, and low stakeholder awareness) describes governance and cultural impediments [46]. The findings indicate that regulatory instruments (e.g., mandates for minimum recycled content in public procurement), combined with targeted awareness campaigns and stakeholder platforms that encourage public-private-academic partnerships, can shift norms and create early markets for recycled materials. Importantly, SEM indicates interdependence between clusters: improving regulatory clarity is likely to reduce perceived financial risk, while strengthened technical systems (data/standards) can increase market confidence—thus interventions should be sequenced and coordinated rather than isolated.

The interplay between institutional, economic and technological barriers identified here aligns with socio-technical transition theory—specifically the Multi-Level Perspective (MLP) framework [47]. MLP posits that transitions emerge from interactions across niche innovations, socio-technical regimes, and landscape pressures. In our results, technological and informational improvements (niche-level enablers such as BIM-enabled traceability and EPD adoption) must align with regime-level change (regulatory reform and procurement incentives) to enable broader CE uptake. The SEM evidence for strong interdependencies among barrier clusters supports a systems view: piecemeal interventions will be less effective than coordinated packages that simultaneously address techno-infrastructural capacity and regulatory-economic incentives.

**Fig 4:**
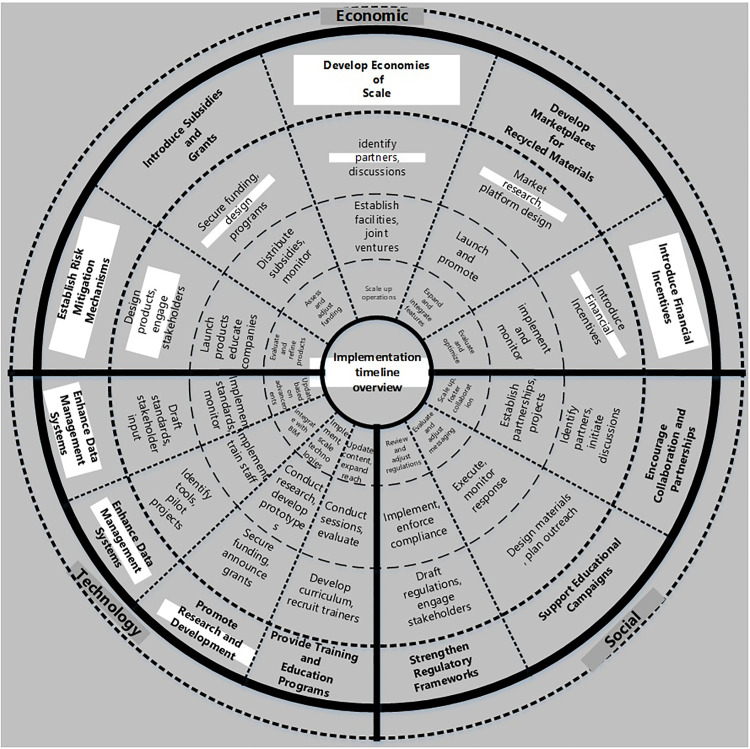
Summary of the Recommendations (short, medium, and long terms) of Different Main Barriers of the Study.

**Fig 5:**
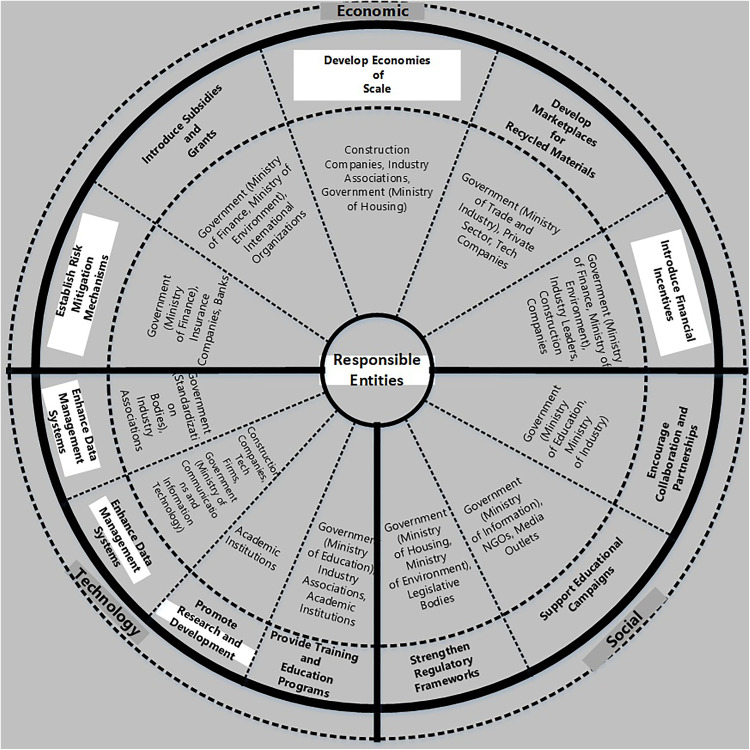
Summary of the Responsible Entities of Different Main Barriers of the Study.

In Table 6, the detailed implementation strategy categorizes the recommendations by barrier type (economic, technological, and social), outlines the steps (Procedures) and timelines for each recommendation, and identifies the responsible entities. This approach facilitates a collaborative effort to overcome the barriers and promote the adoption of CE practices in the Egyptian construction industry. Finally, Figs 4 and 5 show the summary of recommendations (short, medium, and long-term) and the responsible entities of different main barriers of the study, respectively (from the authors’ point of view).

**Table 6:** Implementation timeline overview.

Barrier Type	Recommendation	Short-Term (within one year)	Medium-Term (more than one year and less than three years)	Long-Term (more than three years and less than five years)	Responsible Entities
Economic	Introduce Financial Incentives	Design incentive programs	Implement and monitor	Evaluate and optimize	Government (Ministry of Finance, Ministry of Environment), Industry Leaders, Construction Companies
	Develop Marketplaces for Recycled Materials	Market research, platform design	Launch and promote	Expand and integrate features	Government (Ministry of Trade and Industry), Private Sector, Tech Companies
	Develop Economies of Scale	Identify partners, discussions	Establish facilities, joint ventures	Scale up operations	Construction Companies, Industry Associations, Government (Ministry of Housing)
	Introduce Subsidies and Grants	Secure funding, design programs	Distribute subsidies, monitor	Assess and adjust funding	Government (Ministry of Finance, Ministry of Environment), International Organizations
	Establish Risk Mitigation Mechanisms	Design products engage stakeholders	Launch products, educate companies	Evaluate and refine products	Government (Ministry of Finance), Insurance Companies, Banks
Technological	Standardization of Practices	Draft standards, stakeholder input	Implement standards, monitor	Update based on advancements	Government (Standardization Bodies), Industry Associations
	Enhance Data Management Systems	Identify tools, pilot projects	Implement, train staff	Optimize, and integrate with BIM	Construction Companies, Tech Firms, Government (Ministry of Communications and Information Technology)
	Promote Research and Development	Secure funding, announce grants	Conduct research, develop prototypes	Implement, scale technologies	Government (Research Councils), Private Sector, Academic Institutions
	Provide Training and Education Programs	Develop curriculum, recruit trainers	Conduct sessions, evaluate	Update content, expand reach	Government (Ministry of Education), Industry Associations, Academic Institutions
Social	Strengthen Regulatory Frameworks	Draft regulations engage stakeholders	Implement, and enforce compliance	Review and adjust regulations	Government (Ministry of Housing, Ministry of Environment), Legislative Bodies
	Support Educational Campaigns	Design materials, plan outreach	Execute, monitor response	Evaluate and adjust messaging	Government (Ministry of Information), NGOs, Media Outlets
	Encourage Collaboration and Partnerships	Identify partners, initiate discussions	Establish partnerships, projects	Scale up, foster collaboration	Government (Ministry of Education, Ministry of Industry),

The EFA results group the 10 retained barriers into three factors (economic, technological, and social). The highest-loading items were B1 (low-cost virgin materials), B38 (data quality and accessibility), and B31 (inadequate regulatory oversight), indicating that economic competitiveness, informational infrastructure, and governance are the most critical challenges. SEM confirmed the interdependence of these categories (economic, regulatory and technological factors influence one another), suggesting that addressing one barrier may indirectly mitigate other constraints. The SEM model exhibited acceptable fit (RMSEA = 0.065; CFI = 0.974), supporting the validity of the modeled relationships.

Comparison with prior studies: The dominance of economic and institutional barriers in our Egyptian sample is consistent with findings in several emerging-economy studies [16,25], which similarly report financial and regulatory hurdles as principal constraints. By contrast, some European studies [6,7] place relatively more emphasis on design standards, market instruments, and circular business models, reflecting more advanced policy frameworks and mature secondary markets. Studies from China and the USA [4,15] highlight technological innovations and scale efficiencies but also note that policy alignment remains a key determinant. Our results therefore corroborate a pattern whereby countries at different stages of institutional development present different barrier profiles, underscoring the need for context-sensitive policy design.

## 5. Conclusions, limitations, and future studies

To satisfy the main goals of the research, the following processes are the core of the manuscript. The first is to identify Key Barriers by survey and literature review to pinpoint the primary barriers to CE adoption in the Egyptian construction sector. Secondly, employ EFA and SEM to analyze the impact and interrelationships of these barriers, providing a robust framework for understanding their complexities. The third step is proposing targeted strategies to mitigate these barriers, including economic incentives, technological advancements, and regulatory reforms. Finally, emphasizes the critical role of collaboration among government entities, industry leaders, and academic institutions in driving the adoption of CE practices. The fit qualitative and quantitative analyses that were created, the research guides us for some main conclusions to draw the adoption framework of implementing CE in the construction industry, which are:

1. The effectiveness of the policies can be enhanced by a structured, time-bound approach—comprising short-term design, medium-term implementation, and long-term optimization facilitates the systematic dismantling of economic, technological, and social barriers to sustainable development.
2. Sharing of financial incentives, subsidies, and risk mitigation mechanisms significantly lowers entry barriers, promotes investment in sustainable practices, and accelerates the adoption of CE principles.
3. Establishing uniform standards, enhancing data infrastructure, and promoting research and development are essential for fostering interoperability, scalability, and continuous technological advancement across the construction and recycling sectors.
4. The implementation of targeted training programs and educational initiatives is vital for equipping stakeholders with the necessary skills and knowledge to support and sustain technological and regulatory transformations.
5. Robust legal frameworks, complemented by public awareness campaigns and collaborative partnerships, are instrumental in cultivating a culture of compliance, accountability, and shared responsibility among stakeholders.
6. The involvement of diverse actors—including government agencies, industry leaders, academic institutions, and civil society ensures that policy design and implementation are inclusive, context-sensitive, and resilient to systemic shocks.
7. Embedding feedback loops and evaluation mechanisms within each phase of implementation enables dynamic adjustment, ensures relevance over time, and supports evidence-based decision-making.

From a theoretical standpoint, this study enriches the academic discourse on CE implementation in developing nations by presenting a validated framework that maps out key barriers and their interconnections. It deepens the understanding of how structural and contextual challenges shape the adoption of CE principles within the construction sector. On the practical front, the research offers concrete guidance for policymakers, industry professionals, and researchers. The proposed strategies—tailored to specific stakeholder roles—provide a clear and actionable pathway for advancing CE integration, with particular relevance to the Egyptian construction industry.

This study assesses the degree of adoption of CE in the CDW sector, mainly in Egypt. However, the study covered many main points, but still has some limitations that need to be mentioned in future research. These points can be summarized as follows:

1. The questionnaire should be divided into different construction industry branches and must be regional to cover all of the Middle East. Actually, the case study in this research covered many cities in Egypt but needs to be expanded to cover many other regions.
2. Increase the general data of respondents, and sampling size by considering some other stakeholders and economists.
3. Ensuring that the respondents are working on all types of construction projects. By examining how factors such as project scale, scope, and complexity affect the implementation of CE practices, future research can provide tailored recommendations and strategies for different project contexts.

Furthermore, the scientific rigor of the methodologies employed enhances the credibility and validity of the study’s conclusions, thereby contributing to the advancement of knowledge in the field of sustainability and CE practices within the construction industry.
